# Research on an Intelligent Grading Method for Beef Freshness in Complex Backgrounds Based on the DEVA-ConvNeXt Model

**DOI:** 10.3390/foods14244178

**Published:** 2025-12-05

**Authors:** Xiuling Yu, Yifu Xu, Chenxiao Qu, Senyue Guo, Shuo Jiang, Linqiang Chen, Yang Zhou

**Affiliations:** 1 College of Information and Technology, Jilin Agricultural University, Changchun 130118, China; yuxiuling@jlau.edu.cn (X.Y.); yifuxu@mails.jlau.edu.cn (Y.X.); qcx@mails.jlau.edu.cn (C.Q.); 20251663@mails.jlau.edu.cn (S.G.); 2 College of Food Science and Engineering, Jilin Agricultural University, Changchun 130118, China; whzhang@jlau.edu.cn; 3 College of Biological and Agricultural Engineering, Jilin University, Changchun 130022, China; lqchen23@jlu.edu.cn

**Keywords:** feature extraction enhancement, beef quality evaluation, background replacement, dynamic non-local attention mechanism, enhanced deep convolutional module

## Abstract

This paper presents a novel DEVA-ConvNeXt model to address challenges in beef freshness grading, including data collection difficulties, complex backgrounds, and model accuracy issues. The Alpha-Background Generation Shift (ABG-Shift) technology enables rapid generation of beef image datasets with complex backgrounds. By incorporating the Dynamic Non-Local Coordinate Attention (DNLC) and Enhanced Depthwise Convolution (EDW) modules, the model enhances feature extraction in complex environments. Additionally, Varifocal Loss (VFL) accelerates key feature learning, reducing training time and improving convergence speed. Experimental results show that DEVA-ConvNeXt outperforms models like ResNet101 and ShuffleNet V2 in terms of overall performance. Compared to the baseline model ConvNeXt, it achieves significant improvements in recognition Accuracy (94.8%, a 6.2% increase), Precision (94.8%, a 5.4% increase), Recall (94.6%, a 5.9% increase), and F1 score (94.7%, a 6.0% increase). Furthermore, real-world deployment and testing on embedded devices confirm the feasibility of this method in terms of accuracy and speed, providing valuable technical support for beef freshness grading and equipment design.

## 1. Introduction

Beef is a popular and nutritious food, but its quality deteriorates during storage and sale due to lipid oxidation, protein degradation, and microbial growth [1]. Current freshness grading methods, such as Total Volatile Basic Nitrogen (TVB-N) [2], Thiobarbituric Acid (TBA) [3], and Total Viable Count (TVC) [4], are destructive, time-consuming, and often require laboratory testing, which can alter the beef’s quality and fail to provide real-time, non-destructive detection. Sensory evaluation, though common, is subjective, influenced by individual differences, and lacks precision in detecting subtle changes in color and texture. Therefore, an efficient, intelligent, time-saving, and objective method to assess beef freshness is urgently needed for food safety, consumer health, and reducing economic losses.

Recent studies have explored the use of machine learning and sensor-based methods to assess the quality of meat and food, including digital image processing, electronic noses, and spectroscopy. For instance, Shtepliuk et al. [5] employed an electronic nose integrated with machine learning models to evaluate pork samples. They successfully distinguished fresh meat from meat with a urine odor, achieving 96.5% sensitivity, 95.3% specificity, and an overall accuracy of approximately 93.5%. Moreover, the model was able to differentiate between fresh meat, cooked meat stored for 1–2 days, and meat aged for 3–31 days. Jia, W. et al. [6] integrated an electronic nose with a Support Vector Machine (SVM) to assess the freshness and spoilage of beef samples. The model effectively distinguished between fresh and spoiled samples with high reliability. Sungho Shin et al. [7] used diffuse reflectance spectroscopy in the 480–920 nm range, combined with deep spectral networks and myoglobin data, to assess beef freshness based on pH variations over the storage period. In 78 samples over 17 days, spectroscopy alone achieved an accuracy of 83.6%. Incorporating myoglobin data increased accuracy to 91.9%, with an AUC of approximately 0.958. Arias, E et al. [8] employed a portable VIS-NIR spectroscopic tool (350–2500 nm) to monitor the freshness of packaged pork loin during storage. Using Partial Least Squares Regression, they predicted color and microbial indicators, successfully classifying samples into edible and spoiled categories with over 90% accuracy. However, the machine learning, sensor, and spectroscopic techniques used in these studies face several challenges for beef freshness grading. These include high equipment costs, sensitivity to environmental conditions, time-consuming data collection and preprocessing, limited sensor responsiveness to subtle changes, the need for manual feature extraction, and poor generalizability, all of which hinder their reliable application in practical settings.

Deep learning, particularly Convolutional Neural Networks (CNNs), with their feature extraction capabilities, has outperformed traditional image processing and machine learning methods in advancing computer vision applications in food quality research, especially in meat quality detection and automated freshness grading. Elangovan et al. [9] developed lightweight convolutional networks, ConvNet-18 and ConvNet-24, for binary classification of beef freshness and spoilage, achieving accuracy of 99.4% and 96.6%, respectively. Huang et al. [10] used a computer-vision-based method to automatically identify pork primal cuts (ham, loin, belly, etc.), advancing automation in meat categorization. Song et al. [11] employed a computer-vision-based convolutional neural network model (including MobileNetV3_Small) to automatically identify pork cut types and freshness, achieving an impressive 98.59% classification accuracy. Liu et al. [12] employed the VGG-16 architecture in combination with visible light images to classify pork freshness across varying storage durations, achieving an accuracy of 95.8%. Syaukani et al. [13] developed a hybrid model combining ResNet-50 and SVM, achieving 100% classification accuracy for both beef and pork. Xi et al. [14] proposed a novel non-destructive and real-time YOLO-NF model for discriminating normal and frozen-thawed beef using deep learning techniques, achieving a mean average precision of 98.6% and significantly outperforming YOLOv7, YOLOv5, and YOLOv8 models.

**Figure 1:**
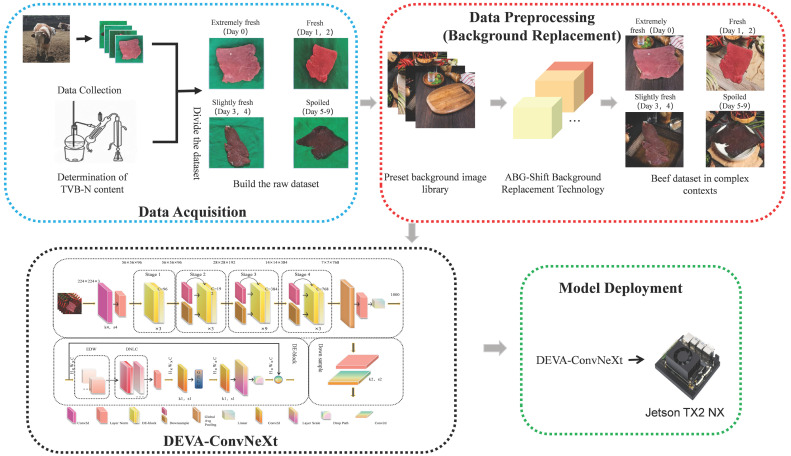
Implementation process of beef freshness grading technology based on the DEVA-ConvNeXt model. The blue dashed line indicates data collection and dataset division based on TVB-N; the red dashed line represents data sample augmentation using background replacement technology; the black dashed line refers to model optimization, training, and validation; and the green dashed line represents model deployment and real-world testing.

Although deep learning shows potential in food quality assessment [15], its practical application faces several challenges. First, existing methods rely on high-quality image data. Complex backgrounds and lighting variations affect model accuracy, reducing the ability to generalize to different environments [16]. Second, studies often use small datasets, and the limited variety in the datasets makes it difficult for models to handle real-world scenarios effectively, especially when dealing with different meat cuts, aging processes, and environmental conditions. Additionally, data collection is costly, limiting model applicability. Some studies depend on expensive equipment, such as spectral data and special labels, which are not commonly used in production environments [17]. Furthermore, previous models often fail to capture the subtle differences in freshness that are crucial for accurate food quality assessment, as they are heavily affected by noise from heterogeneous data sources, leading to lower classification accuracy. This study proposes the DEVA-ConvNeXt network, combined with background replacement technology. Enhancing multi-scale feature extraction significantly improves classification accuracy. The data collection process is optimized, reducing reliance on costly equipment and increasing dataset diversity. This makes the model more robust and generalizable both in academic research and practical applications. The method provides crucial technical support for the development of low-cost portable beef freshness grading devices and intelligent food quality inspection systems. As shown in Figure 1, this illustrates the main research focus and the implementation process of beef freshness grading technology based on the DEVA-ConvNeXt model.

## 2. Data Acquisition and Dataset Construction

### 2.1. Image Collection

The experimental data in this study were obtained from aged chilled beef samples, sourced from a local vendor in Beian City, Heilongjiang Province. To minimize the impact of the meat backlog on classification, beef rib cuts produced at 5:11 AM on the same day were selected and sliced. Image acquisition and chemical indicator analysis [18] of the beef samples were performed simultaneously to ensure accurate grading of beef freshness.

The data acquisition device used in the experiment was the HONOR Magic V2, with an image resolution of 3072 × 4096 pixels, saved in JPG format. Images were captured daily at 7:00 AM under natural light and at room temperature (20–25 °C), with a standardized green screen background, and at a vertical distance of 25–30 cm from the beef samples. A total of 1523 images were collected as raw data. Additionally, TVB-N samples were collected daily at 9:00 AM for chemical analysis. Image capture and TVB-N content detection of beef samples were conducted simultaneously over a 10-day period to ensure accurate grading of beef freshness. After capturing the freshest beef images, the slices were neatly arranged in a clean, dry food preservation box and stored in a refrigerator at 0–4 °C to prevent sticking and contamination. Other items were removed from the fridge to avoid odor interference. All chemical reagents used in the experiment were of analytical grade, ensuring the accuracy and reliability of the results.

### 2.2. Determination of TVB-N Content

TVB-N is established by national standards as the sole chemical indicator effectively characterizing meat freshness, as detailed in China’s national standard GB 2707-2016 “National Food Safety Standard for Fresh (Frozen) Meat and Poultry Products” [19]. In this experiment, TVB-N content in beef samples was determined using the semi-micro nitrogen determination method, in strict accordance with the procedures outlined in the national food safety standard GB 5009.228-2016 “Determination of Total Volatile Basic Nitrogen in Foods” [20]. A beef sample was randomly selected, divided into three equal portions, and each portion was analyzed for TVB-N content. The final chemical indicator content at each experimental time point was represented by the average of the three measurements. The procedure began with thoroughly homogenizing the beef sample. It was then soaked for 30 min. Next, the extract was boiled, and the condensed vapor was collected. Finally, titration was performed using a standardized hydrochloric acid solution.

As shown in Table 1, this is the TVB-N content-based grading system for beef freshness.

As shown in Table 1, this grading system classifies beef freshness based on TVB-N content. TVB-N is a key indicator of meat freshness, with its level increasing as storage time lengthens, reflecting changes in beef quality. The TVB-N content is divided into three categories: Grade 1 Freshness (Fresh) for TVB–N ≤ 15 mg/100 g, Grade 2 Freshness (Slightly Fresh) for 15–25 mg/100 g, and Spoiled for TVB–N ≥ 25 mg/100 g.

**Figure 2:**
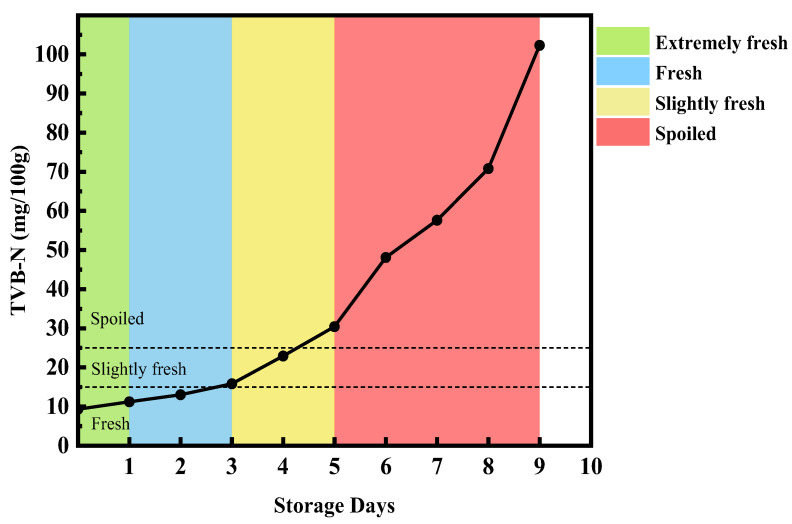
Schematic diagram of beef freshness grading based on TVB-N content over different days.

As shown in Figure 2, this schematic diagram illustrates the beef freshness grading based on TVB-N content over different days. As beef is stored, an increase in TVB-N content indicates a decline in freshness. In this study, the initial TVB-N value of beef on day 0 was 9.3 mg/100 g, with subsequent measurements on days 1 to 9 showing values of 11.2, 13.0, 15.8, 22.9, 30.4, 48.1, 57.6, 70.8, and 102.3 mg/100 g, respectively. According to the TVB-N content freshness grading chart in Table 1, the beef freshness classification was as follows: Day 0, extremely fresh; Days 1 to 2, fresh; Days 3 to 4, slightly fresh; Days 5 to 9, spoiled.

### 2.3. Dataset Construction

#### 2.3.1. Dataset Splitting

In accordance with the beef freshness grading standards outlined in the Chinese national standard GB 2707-2016 “National Food Safety Standard for Fresh (Frozen) Meat and Poultry Products,” and with guidance from experts at the College of Food Science, Jilin Agricultural University, a total of 1523 beef samples were graded and annotated. A secondary screening process excluded samples with unclear characteristics, atypical colors, or excessive similarity, resulting in a final set of 1200 valid beef images. These images were then organized into ten folders based on their respective freshness days, creating a self-constructed dataset for the study.

**Figure 3:**
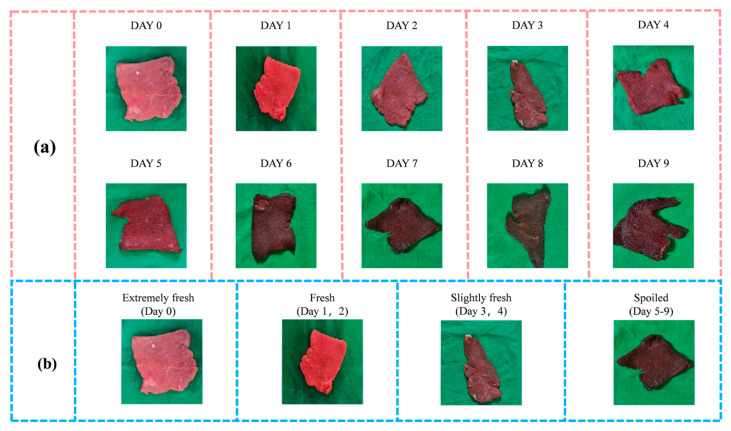
Example of the raw image dataset of beef freshness samples. (**a**) Images of beef samples on different storage days; (**b**) images of beef samples for four freshness levels.

The dataset is divided as follows: Day 0 extremely fresh with 109 samples, Days 1–2 fresh with 243 samples, Days 3–4 slightly fresh with 266 samples, and Days 5–9 spoiled with 582 samples. After completing the freshness level classification, this study used individual images as the unit for classification. Within each level, beef sample images were divided in a ratio of 8:1:1. As shown in Figure 3, the raw data of the beef samples are presented, with (a) displaying the images of beef samples taken on different days, and (b) showing the images of beef samples categorized by freshness levels. The distribution of freshness levels in the collected beef freshness sample image dataset is shown in Table 2.

#### 2.3.2. Enhanced Dataset with Background Replacement Based on ABG-Shift Technology

**Figure 4:**
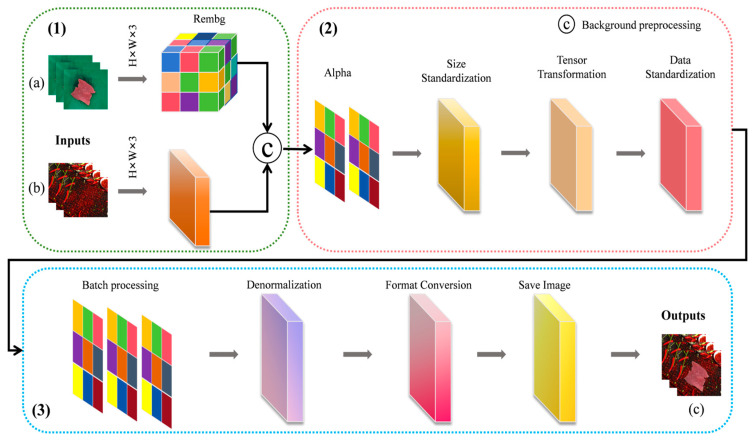
Implementation process of background replacement technology based on ABG-Shift. (**a**) Beef sample image with the original green screen background; (**b**) image with a complex background; (**c**) beef sample image with a complex background after replacement.

In practice, collecting beef sample data is both time-consuming and labor-intensive. Additionally, due to the complexity of scene setup, efficiently obtaining diverse backgrounds remains a significant challenge. To address these issues and improve the efficiency and flexibility of data acquisition, this study adopts a background replacement strategy. First, original data are captured in a controlled green-screen environment. Then, background replacement technology is applied to seamlessly replace the original background with various real-world settings, such as supermarket shelves, cold storage, dining tables, and cutting boards. This approach helps in the rapid generation of diverse datasets. As shown in Figure 4, the process of background replacement for beef sample images using Alpha-Background Generation Shift (ABG-Shift) technology is illustrated.

As shown in Figure 4, the implementation of the background replacement technology, ABG-Shift, is based on the U^2^-Net deep learning model from the rembg library [21], which can automatically recognize and segment the main objects in the image. The specific implementation process is as follows:

**(1)** The system reads the original foreground image and converts it to RGB format. Then, the rembg library is used to extract the foreground edge features of the input image while generating an RGB image with an Alpha transparency channel. This allows for precise, pixel-level foreground segmentation.

**(2)** A background image is randomly selected from a preset background library and resized to match the dimensions of the beef sample in the foreground image. Using Alpha channel blending techniques, the beef foreground is then merged into the new background, achieving background replacement and lighting/shadow matching. After obtaining the image, it is standardized to a size of 224 × 224 × 3 and converted to tensor format, followed by data normalization.

**(3)** Finally, a de-normalization and format conversion module is designed to restore the normalized tensor back to a visible image format for saving.

The pseudocode for the Algorithm of the ABG-Shift method is Algorithm 1 as follows:
**Algorithm 1 Alpha-Background Generation Shift**1: Input: Foreground image $I_{fg}$, mask generated by rembg, Background pool B, Number of synthetic samples $N_{bg}$
2: Output:Synthetic set {$I^{\left(1\right)}$,…, $I^{\left(N_{bg}\right)}$}3:    for i = 1 to $N_{bg}$ do4:       $I_{cut}$ ← Rembg ($I_{fg}$)5:         select background $B_{i}$~B
6:         resize $B_{i}$ to match $I_{cut}$
7:       apply random scale/rotation/flip on $I_{cut}$
8:       $I_{cut}$ ← ReinhardColorTransfer ($I_{cut},B_{i}$)9:       use alpha mask to blend $I_{col}$ with $B_{i}$
10:        $I_{blend}^{\left(i\right)}$ = AlphaBlending ($I_{col},B_{i}$)11:        $I^{\left(i\right)}$ ← RandomJPEG ($I_{blend}^{\left(i\right)}$)12:      apply random Gaussian/uniform noise 13:      save metadata:14:     {fg_id, bg_id, transforms, seed}15:   end for16:   return {$I^{\left(1\right)}$,…, $I^{\left(N_{bg}\right)}$}

**Figure 5:**
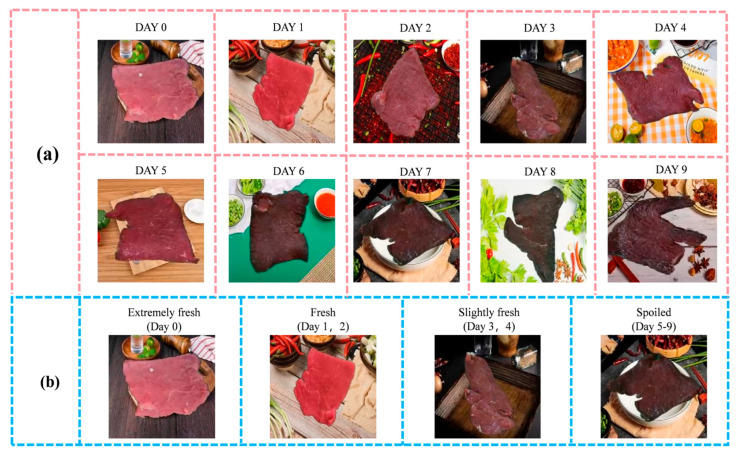
Beef freshness sample image dataset with complex backgrounds. (**a**) Images of beef samples from different days. (**b**) Images of beef samples at different freshness levels.

By following the steps outlined above, a composite beef image with a complex background (Figure 4c) is generated by combining a clean background beef image (Figure 4a) and a complex background image (Figure 4b), which is then used for beef freshness level recognition. This method reduces image capture time to about 10 s per image, saving time on scene selection, setup, and repeated shooting, thus improving efficiency. It provides the model with complex background data, enriching the dataset’s diversity, overcoming the limitations of a single background, and enhancing the model’s adaptability, robustness, and generalization in real-world settings. The background images come from two main sources: some were collected from public networks, showcasing real-world scenes, while others were captured on-site at the experimental location, ensuring alignment with actual collection conditions. In total, 100 complex background images were gathered. Figure 5 shows the beef sample images after background replacement.

As shown in Figure 5, after applying the background replacement technique, the integrity and color-texture features of the beef samples are preserved. The clean-background samples in Figure 3 are replaced with images featuring more complex backgrounds that approximate real-life scenarios. This approach not only increases the diversity of the training data but also reduces the risk of overfitting to a single background. By using this method, the model learns more robust feature representations, demonstrating superior generalization performance in practical applications and making the trained model better suited for beef freshness grading tasks.

## 3. Construction of Beef Freshness Grading Model Based on DEVA-ConvNeXt

In beef freshness grading, key challenges include background interference and subtle intra-class variations, particularly in features like color and texture. These issues make it difficult for traditional methods to accurately extract and differentiate freshness levels. To address these, we propose the DEVA-ConvNeXt model, based on the ConvNeXt [22] architecture. This model is specifically designed to focus on phenotypic traits (such as color and texture), overcoming background noise and subtle differences, resulting in more precise freshness grading.

**Figure 6:**
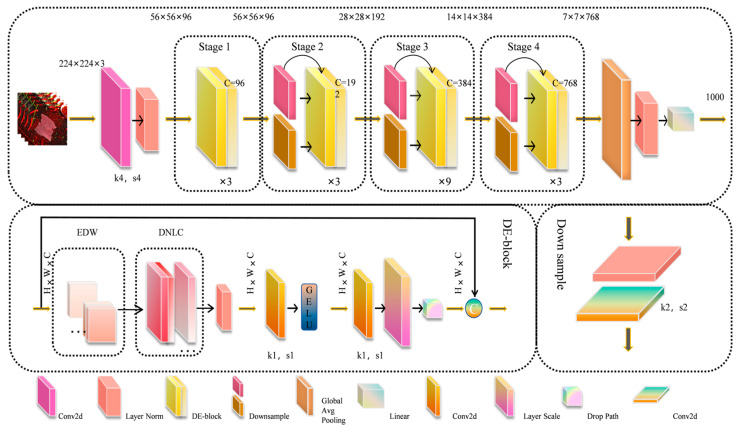
Overall architecture of the DEVA-ConvNeXt model.

To address the challenges posed by background interference and the difficulty in capturing the diverse features of beef, this study introduces the Dynamic Non-Local Coordinate Attention (DNLC) mechanism. DNLC effectively models key information such as target location, global context, color, and texture, while suppressing background interference and improving grading accuracy. However, DNLC has limitations in deep feature extraction and fusion across multiple scales and levels. To enhance the model’s performance, an Enhanced Depth-wise Convolution (EDW) module is proposed. EDW integrates large receptive field convolutions with multi-level depth-wise convolutions, enhancing the representation of complex textures and color features, and overcoming the limitations of feature extraction and fusion. For the training strategy, Varifocal Loss (VFL) [23] is introduced as a replacement for the traditional cross-entropy loss function [24]. VFL applies asymmetric weighting to positive and negative samples, focusing training on high-quality samples, optimizing gradient signal-to-noise ratio, and accelerating model convergence. As shown in Figure 6, EDW and DNLC together form a novel DE-block.

As shown in Figure 6, the DEVA-ConvNeXt consists of four stages, with 3, 3, 9, and 3 DE-blocks stacked in each stage. Downsampling modules, composed of LayerNorm and convolutions with a stride of 2, connect the stages. At the end of the network, global average pooling, layer normalization, and a fully connected layer (Linear) are used for classification tasks. By integrating these improvements, DEVA-ConvNeXt achieves more efficient and accurate beef freshness grading under complex backgrounds, significantly enhancing sensitivity to color and texture features while effectively suppressing background interference. The model excels in accuracy, convergence speed, and generalization ability, while also balancing computational efficiency and performance, showcasing its potential for use in resource-constrained automated quality inspection systems.

### 3.1. ConvNeXt Model

**Figure 7:**
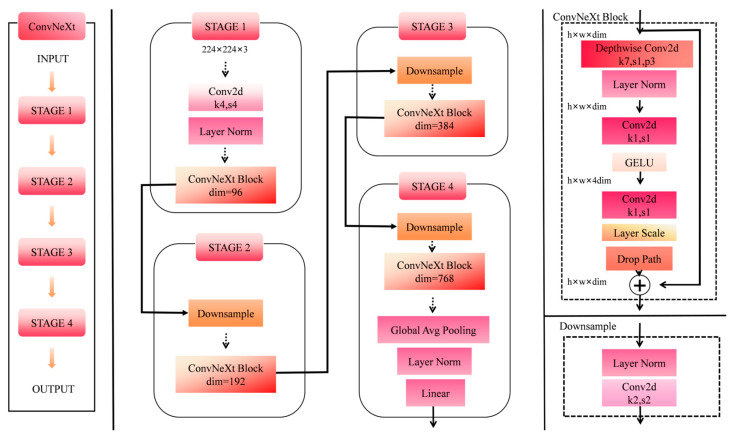
Structure diagram of the ConvNeXt model.

To effectively distinguish subtle features in beef texture and color across different freshness levels, this study selects the ConvNeXt model, known for its excellent feature extraction capabilities. The model efficiently captures visual information such as beef color, texture, and marbling patterns. Traditional convolutional networks are limited in their feature extraction abilities and struggle with fine-grained classification tasks. ConvNeXt, introduced by Liu et al. at Facebook AI Research (FAIR), incorporates innovations from the Swin Transformer [25], modernizing standard convolutional networks and achieving significant results in image classification and object detection. After comparing several mainstream models, ConvNeXt was selected as the benchmark for this study. For tasks like beef freshness grading, which require high representation power, ConvNeXt offers clear advantages. Its core module, the ConvNeXt Block, combines large-kernel depth-wise separable convolutions with an inverted bottleneck structure, replacing traditional residual blocks and enhancing feature learning capabilities. As shown in Figure 7, this is the structure diagram of the ConvNeXt model.

However, traditional ConvNeXt still has limitations in beef freshness grading under complex backgrounds, including (1) limited suppression of interference information, (2) weak multi-scale feature fusion and insufficient capture of detailed features, and (3) suboptimal training optimization strategies. Therefore, this study further investigates and improves the model to enhance grading accuracy.

### 3.2. DNLC Attention Mechanism

This section introduces the Dynamic Non-Local Coordinate (DNLC) module proposed in this study. The module addresses the challenge of capturing the diverse surface features of beef in the freshness grading task. It does so by leveraging three parallel branches: multi-scale convolution, lightweight non-local modeling, and color statistics. These branches work together to overcome the issues caused by complex background interference and fixed receptive field limitations.

**Figure 8:**
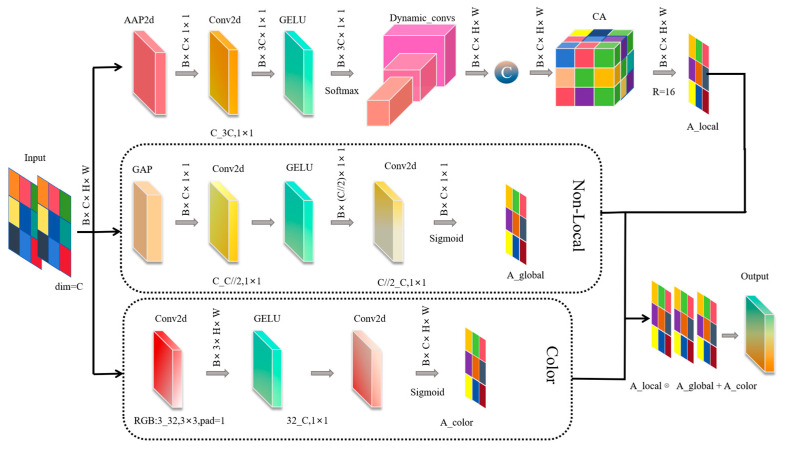
Structure diagram of the DNLC attention mechanism.

In beef freshness grading tasks, background interference and feature capture are key challenges. Conventional attention mechanisms, constrained by fixed receptive fields, struggle to capture the diverse features of the beef surface simultaneously. Moreover, when dealing with complex backgrounds, attention tends to shift towards irrelevant regions, resulting in insufficient extraction of key features, which affects grading accuracy. To address this challenge, this study introduces the DNLC module. The structure diagram of the DNLC attention mechanism is shown in Figure 8.

The module consists of three parallel branches, each focusing on multi-scale detail modeling, global context modeling, and color feature extraction, with complementary enhancement achieved through weighted fusion. Initially, DNLC begins with a dynamic multi-scale convolution branch. The branch first dynamically generates a set of weights using a lightweight weight generator with a 1 × 1 convolution (reduction = 1) and GELU activation, followed by normalization with the Softmax function and a temperature parameter τ=0.5. The calculation formula is shown in Equation (1).

(1) $$\omega =\sigma \left(C_{1\times 1}\left(\delta (\gamma (X))\right)\right)$$

Here, ω represents the dynamic weights, σ is the Softmax function, $C_{1\times 1}$ denotes the 1 × 1 convolution, δ is the GELU activation function, γ refers to adaptive average pooling, and X is the input feature map. These weights are passed through three parallel Depthwise Separable Convolutions [26] with kernel sizes of 3 × 3, 5 × 5, and 7 × 7, allowing adaptive receptive field adjustment and multi-scale local feature extraction. The fused features are processed through an enhanced coordinate attention module, with a channel compression ratio of reduction = 16. This module accurately locates key beef features and generates the local context attention map $A_{l}$. The calculation formula is as follows:

(2) $$F_{1}=\omega_{3}\cdot DW_{3\times 3}(X_{1})+\omega_{5}\cdot DW_{5\times 5}(X_{1})+\omega_{7}\cdot DW_{7\times 7}(X_{1})$$

(3) $$A_{l}=\beta \left(\delta (C_{k\times k}(F_{1}))\right)$$

where $F_{1}$ represents the fused feature map, $X_{1}$ is the input feature map, ${DW}_{n\times n}\left(X_{1}\right)$ denotes feature extraction, $\omega_{3}$, $\omega_{5}$, and $\omega_{7}$ represent the dynamic weights, β is the Sigmoid activation function, δ is the GELU activation function, and $C_{k\times k}$ represents the standard convolution layer.

To establish a global view, a lightweight parallel Non-Local branch is used to process the input features directly. This branch first captures global context information through Global Average Pooling, then efficiently establishes global dependencies between features using a Multilayer Perceptron, ultimately generating the global context attention map $A_{g}$. The calculation process is shown in Equation (4).

(4) $$A_{g}=\beta \left(\delta \left(C_{2D}(\lambda (X_{2}))\right)\right)$$

where β is the Sigmoid activation function, δ is the GELU activation function, $C_{2D}$ represents the Multilayer Perceptron (MLP) structure, λ is the Global Average Pooling (GAP), and $X_{2}$ is the input feature. According to the national standard GB/T 29392-2022 “Grading of Livestock and Poultry Meat Quality” [27], as beef deteriorates from fresh to spoiled, its surface undergoes a noticeable color change, shifting from bright red to dark brown. Therefore, color information serves as a key indicator for determining beef freshness. Considering the importance of color in beef freshness classification, a third parallel color statistics enhancement branch is used to extract the RGB channels of the input features. Through a small convolutional network and the Sigmoid function, key color features are distilled, ultimately generating the color feature map $A_{c}$. The calculation process is shown in Equation (5).

(5) $$A_{c}=\beta \left(SC(X_{RGB})\right)$$

Here, SC refers to the Small Convolutional Network, and $X_{RGB}$ represents the RGB channels of the input features. In the entire attention mechanism, the branches complement each other. The weighted fusion produces the comprehensive attention map, which is applied to the original features to enhance key regions and channels while suppressing background interference. This fused attention map is applied to the original feature map, dynamically weighting the original features to enhance key region and channel features while suppressing background and irrelevant information. The calculation process is shown in Equation (6).

(6) $$F_{A}=A_{l}\cdot A_{g}+A_{c}$$

Here, $F_{A}$ denotes the Final Fusion Attention Map. Integrating the DNLC attention mechanism into the DE-Block of the DEVA-ConvNeXt model significantly enhances the model’s ability to capture multi-scale features of the beef surface while suppressing complex background interference, thereby improving the accuracy of beef freshness grading.

### 3.3. Enhanced Depthwise Convolution Module

After addressing irrelevant information interference and insufficient feature diversity capture with the DNLC attention mechanism in Section 3.2, the model still faces challenges in extracting and fusing multi-scale, multi-level features. To further optimize performance, this section introduces the Enhanced Depthwise Separable Convolution Module (EDW). The module uses a multi-stage depthwise separable convolution structure, combining convolution kernels of different sizes with residual connections to enable layer-by-layer feature extraction.

The implementation process of EDW is as follows:

First, the input feature map passes through a 7 × 7 Depthwise Separable Convolution ${DWConv}_{7\times 7}$ with stride s=1 and grouped channels equal to the input channels. This expands the effective receptive field without changing the spatial resolution, capturing macro-contextual information. The convolution results are then passed through batch normalization (BN) using BN2d and GELU activation, as fixed hyperparameters for the EDW module. The outputs are added to the original input via a residual connection, effectively alleviating gradient vanishing and preserving key information, as shown in Equation (7):

(7) $$O_{1}=\delta \left(\varepsilon \left(DWConv_{7\times 7}(I)\right)\right)+I$$

Here, $O_{1}$ represents the macroscopic feature map processed by a 7 × 7 deep convolution with stride s=1 and the first residual connection, δ denotes the GELU activation function, ε refers to BN, and I is the original input feature map.

The process moves to the fine-grained feature refinement stage. $O_{1}$ undergoes a 3 × 3 Depthwise Separable Convolution (${DWConv}_{3\times 3}$) to extract local edges and detailed features. This stage also incorporates BN and the GELU activation function. Additionally, an optional channel expansion and compression mechanism is applied: when the expansion factor (E) exceeds 1, pointwise convolutions [28] first increase and then decrease the dimensionality to enhance feature representation. In this study, we set E=1 as default. The number of channels is expanded from dim to E×dim, and then compressed back to dim to enhance the expressiveness of intermediate channels while keeping the input and output channel numbers unchanged. Finally, the extracted features are added to $O_{1}$ through a second residual connection, producing the feature map $O_{2}$. The calculation process is as follows:

(8) $$T_{F}=\delta \left(\varepsilon \left(DWConv_{3\times 3}(O_{1})\right)\right)$$

(9) $$E_{F}=PConv_{C}\left(PConv_{E}(T_{F})\right)$$

(10) $$O_{2}=E_{F}+O_{1}$$

Here, $T_{F}$ represents the intermediate features after the 3 × 3 convolution, and $E_{F}$ denotes the features after the expansion and compression process. ${PConv}_{C}$ and ${PConv}_{E}$ refer to the pointwise convolutions for dimensionality expansion and reduction, respectively. $O_{2}$ is the output after the second residual connection.

Finally, in the feature integration stage, $O_{2}$ undergoes another 3 × 3 Depthwise Separable Convolution, followed by BN and GELU to further integrate the information. It then forms a third residual connection with the input. The output is multiplied by a learnable scale factor to adaptively adjust the module’s contribution to the overall features, enabling dynamic feature weighting. The computation is given by Equation (11):

(11) $$I_{F}=\delta \left(\varepsilon \left(DWConv_{3\times 3}(O_{2})\right)\right)+O_{2}$$

(12) $$O_{F}=I_{F}\cdot LSF$$

**Figure 9:**
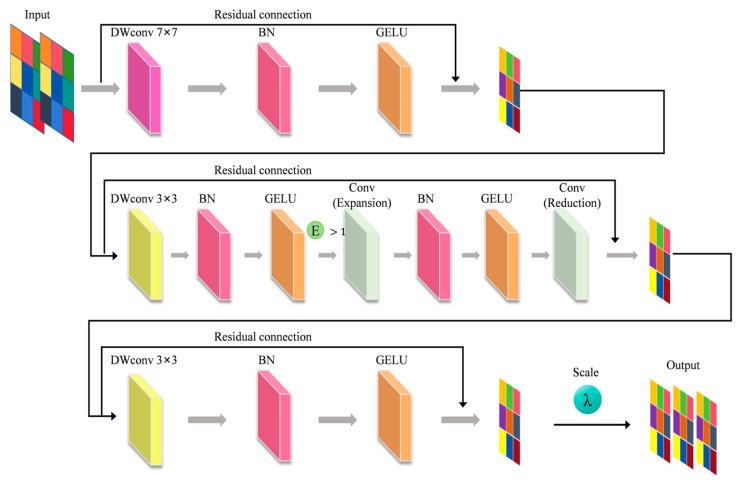
Implementation principle and structure diagram of the EDW module.

Here, $O_{F}$ represents the final output of the module, and $I_{F}$ is the feature map after the last convolution and residual connection. LSF is the learnable scale factor. As shown in Figure 9, the diagram illustrates the structural design and overall workflow of the EDW module.

As shown in Figure 9, the EDW module enhances feature extraction and fusion through five key components: (1) large kernel convolutions for global semantics, (2) 3 × 3 convolutions for refining local details, (3) an expansion–compression structure to boost channel representation, (4) residual connections for stable training, and (5) a scale factor for dynamic weighting. This approach significantly improves the extraction and integration of multi-scale features. In the beef freshness grading task, the module effectively captures key features such as texture and color, enhancing the model’s discriminative power and robustness in complex backgrounds.

### 3.4. Varifocal Loss

Varifocal Loss (VFL) is an improved version of Focal Loss, designed to address the issue of class imbalance during training. Unlike Focal Loss, which suppresses all samples equally, VFL uses an asymmetric weighting strategy. It reduces the weight of negative samples while increasing the weight of high-quality positive samples, focusing training on more discriminative positive examples. This approach helps enhance the model’s accuracy and robustness.

VFL was originally used to train IoU-aware Classification Scores (IACS) for dense object detectors. This metric not only incorporates classification confidence but also integrates localization accuracy, enabling the model to more effectively rank predictions and focus on optimizing high-quality positive samples, thus significantly improving performance.

The description of the calculation process is as follows:

For positive samples (q > 0):

(13) $$VFL(p,q)=q\cdot \alpha \cdot q^{\gamma }\log(p)+(1-q^{\gamma })\log(1-p)$$

For negative samples (q > 0):

(14) $$VFL(p,q)=-(1-q^{\gamma })\log(1-p)$$

Here, p is the predicted IACS by the model. q is the target IoU score. α and γ are hyperparameters in the training process. They adjust the trade-off between localization accuracy and classification confidence.

Since the original VFL was designed for object detection tasks, this study adapts it to the multi-class scenario of beef freshness grading by converting the labels into One-Hot encoding. The original target tensor has the shape N, C, where N is the batch size and C is the number of classes. After conversion, the correct class index for each sample is set to 1.0, with all other positions set to 0.0. After applying One-Hot encoding, VFL naturally distinguishes between the correct class (target = 1) as a positive sample, and the incorrect classes (target = 0) as negative samples. This approach effectively reduces the contribution of easily distinguishable negative samples to the loss, allowing the model to focus more on difficult-to-classify samples and key class features.

In beef freshness grading, VFL improves robustness in complex backgrounds by suppressing the loss weight of background and simple categories. It amplifies the loss of positive and hard-to-distinguish negative samples, enhancing the model’s ability to discern subtle texture and color differences. Targeted gradient updates avoid wasting computational resources, speeding up convergence and improving learning efficiency.

## 4. Experiments and Results Analysis

### 4.1. Experimental Setup and Evaluation Metrics

#### 4.1.1. Experimental Setup

In the experiment, the hardware for model training includes an Intel i7-7820X CPU (3.60 GHz; Intel Corporation, Santa Clara, CA, USA), two TitanXp GPUs (12.0 GB memory; NVIDIA Corporation, Santa Clara, CA, USA), and CUDA 11.0. The operating system is Windows 10, with a software environment configured as Python 3.8 and PyTorch 1.9.0, developed using PyCharm 2024.1.

In model training, the AdamW optimizer is used with a batch size of 8, weight decay of 0.01, and 200 training epochs. Input images are resized to 224 × 224. To enhance training performance and accelerate convergence, a cosine annealing learning rate strategy is applied. Initially, a high learning rate and low momentum help the model escape poor local minima caused by noise. Later, a low learning rate and high momentum enable stable and precise fine-tuning of key features. This dynamic adjustment mechanism significantly improves convergence speed, final accuracy, and robustness against complex disturbances. The experimental parameter settings are as follows, as shown in Table 3.

#### 4.1.2. Model Evaluation Metrics

To evaluate the performance of the DEVA-ConvNeXt model, the classification results are categorized into true positives (TP), false positives (FP), true negatives (TN), and false negatives (FN). The model’s grading performance for each beef freshness level is assessed using accuracy, precision, recall, and F1 score. The detailed calculation formulas for Precision, Recall, Accuracy and F1 are provided in Equations (15)–(18).

(15) $$Precision=\frac{TP}{TP+FP}$$

(16) $$Recall=\frac{TP}{TP+FN}$$

(17) $$F1=\frac{2\cdot Precision\cdot Recall}{Precision+Recall}$$

(18) $$Accuracy=\frac{TP+TN}{TP+FN+FP+TN}$$

### 4.2. Performance Analysis of DNLC Attention Mechanism

To evaluate the performance of the proposed DNLC attention mechanism, we conducted comparative experiments with several representative attention modules, including SE (Squeeze-and-Excitation) [29], SimAM [30], CBAM (Convolutional Block Attention Module) [31], and CA (Coordinate Attention) [32], all of which have been widely validated in recent visual tasks. ConvNeXt serves as the base model, with each attention module integrated at the same block position. The experimental results, shown in Table 4, compare the performance of different attention mechanisms in beef freshness grading under complex backgrounds.

As shown in Table 4, SE and SimAM achieved limited improvements across all metrics, with an F1 score of 89.9% and 89.7%, respectively. Their overall performance was stable but not sufficient to significantly enhance the model’s discriminative ability. CBAM demonstrated balanced performance across the four metrics, with an F1 score of 90.1%, slightly outperforming the previous two. CA excelled in Precision (91.5%) and Recall (90.5%), but its overall performance had certain limitations. In contrast, the proposed DNLC outperformed all attention mechanisms, achieving optimal results in Accuracy, Precision, Recall, and F1 score, all reaching 92.8%. This significant performance boost indicates that DNLC more effectively models the dependencies between global and local features within the ConvNeXt framework, enhancing the model’s robustness and generalization ability in complex environments. Thus, DNLC not only excels in individual metrics but also demonstrates superior overall performance, providing an efficient and reliable attention-enhancement strategy for beef freshness grading.

### 4.3. Ablation Study for Evaluating Model Performance

To systematically evaluate the performance improvement brought by the proposed modification modules to the ConvNeXt model, a series of ablation experiments were conducted using ConvNeXt as the baseline model. The experimental results are shown in Table 5, where the symbol “√” indicates that the modification has been applied, and indicates that it has not. By comparing the performance of the baseline model with different combinations of improvements, the contribution of each module and method to the overall model performance can be clearly analyzed.

As shown in Table 5, introducing the DNLC attention mechanism significantly improved model performance. Accuracy increased from 88.6% to 92.8%, with Precision, Recall, and F1 score each improving by approximately 4%. This indicates that the attention mechanism effectively extracts more discriminative contextual information, enhancing feature representation. Replacing the Depthwise Separable Convolution with EDW further increased Accuracy to 90.2%, with Precision, Recall, and F1 score improving by over 1%, demonstrating the positive impact of the modified convolution structure on fine-grained feature extraction. Using VF Loss as the training loss function also enhanced performance by amplifying the loss weights for positive and hard-to-classify negative samples, improving the model’s ability to distinguish subtle features and accelerating training convergence. When DNLC and EDW were combined, the performance improvements were more pronounced, with Accuracy reaching 94.7%, Precision and Recall at 94.8% and 94.5%, respectively, and F1 score increasing to 94.6%. After integrating Varifocal Loss, the model achieved the best results across all metrics, with Accuracy at 94.8%, Precision and Recall exceeding 94%, and F1 score reaching 94.7%. These results confirm that the synergy of multiple modules ensures stable training, fast convergence, and improved discriminative ability and generalization, validating enhanced effectiveness.

### 4.4. Performance Comparison and Analysis of Different Models

To evaluate the performance of the DEVA-ConvNeXt model in beef freshness grading, we compared it with several popular deep neural networks: AlexNet [33], ResNet50 [34], ResNet101, GoogLeNet [35], MobileNet V2 [36], MobileNet V3 [37], EfficientNet [38], EfficientNet V2 [39], ConvNeXt, and ShuffleNet V2 [40]. All experiments were conducted under the same training conditions using the AdamW optimizer, a batch size of 8, a weight decay of 0.01, and 200 training iterations.

**Figure 10:**
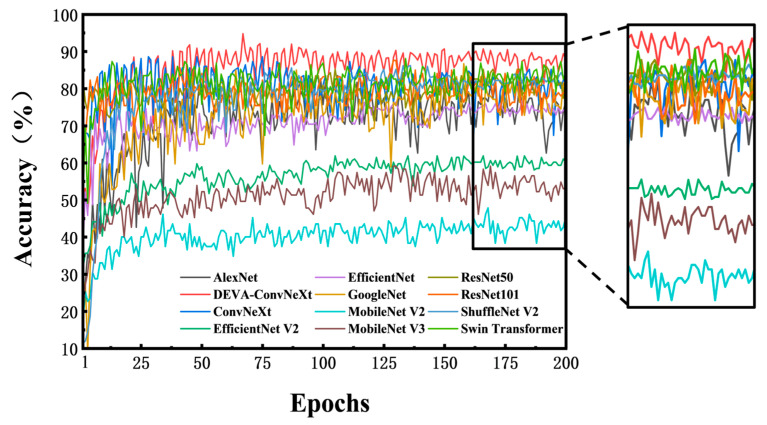
Comparison of validation accuracy across different models.

To visually compare the training performance, convergence speed, and stability of different models under identical training conditions, Figure 10 shows the validation accuracy of each model.

As shown in Figure 10, the DEVA-ConvNeXt model performs excellently throughout training. Its accuracy steadily improves with more iterations, converging quickly. In later stages, the accuracy curve is higher and less volatile, demonstrating better stability and robustness.

**Figure 11:**
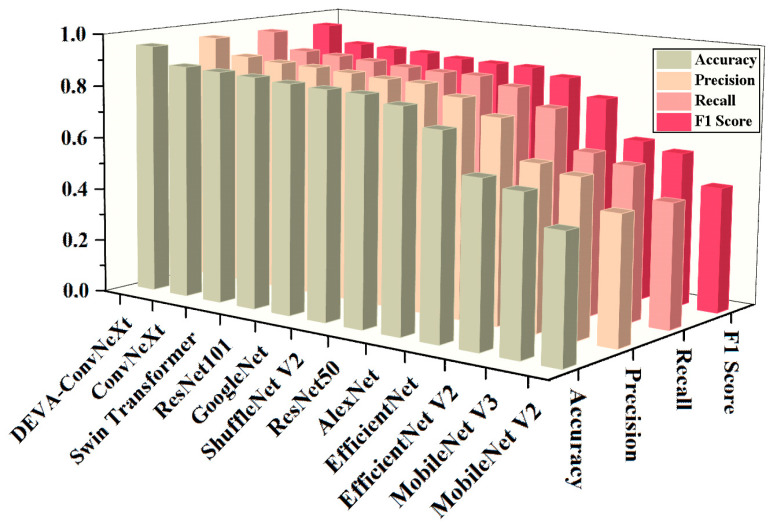
Comparison of the proposed DEVA-ConvNeXt with different models. The *x*-axis represents different beef freshness grading models, the *y*-axis shows the model performance evaluation metrics, and the *z*-axis corresponds to the values of these metrics.

To provide a clear and comprehensive comparison of model performance across key metrics (Accuracy, Precision, Recall, and F1 score), Figure 11 demonstrates the advantages of the proposed model on the validation set in beef freshness grading.

As shown in Figure 11, the performance metrics of the proposed DEVA-ConvNeXt are significantly higher than those of other models, providing preliminary evidence of its advantage in beef freshness classification. To further validate the advantage of the proposed model, numerical results for different models are provided based on Figure 11, as shown in Table 6.

As shown in Table 6, DEVA-ConvNeXt outperforms all other models in key metrics such as classification Accuracy, Precision, Recall, and F1 score on the validation set. The improved DEVA-ConvNeXt model achieves significant gains across all metrics, with accuracy reaching 94.8%, surpassing Swin Transformer (88.1%) and showing a 6.2% improvement over the best-performing ConvNeXt in the original model (88.6%); Precision at 94.8%, up 5.4% from ConvNeXt (89.4%); Recall at 94.6%, a 5.9% increase from ConvNeXt (88.7%); and an F1 score of 94.7%, improving by 6.0% over ConvNeXt (88.7%). In contrast, MobileNet V2, MobileNet V3, EfficientNet, and EfficientNet V2 all show metrics below 80%. This indicates that these models fail to effectively capture the subtle color and texture features of beef surfaces, leading to poor differentiation between beef freshness levels and background, resulting in high false negatives. ResNet50 and ResNet101, with Accuracy rates of 86.0% and 87.6%, respectively, show strong feature extraction ability but are limited in modeling fine texture and color differences. Early models like AlexNet and GoogLeNet, while providing baseline performance, still struggle to capture fine-grained features due to limitations in depth and feature representation. Compared to ShuffleNet V2, DEVA-ConvNeXt shows clear advantages in maintaining high Recall and F1 scores, better reflecting the model’s reliability in classification tasks. The performance metrics of MobileNet V2, MobileNet V3, EfficientNet, and EfficientNet V2 are significantly lower across the board. This is primarily due to the widespread use of depthwise separable convolutions and early aggressive downsampling strategies in these networks, which limit cross-channel information exchange and make it difficult to effectively capture the subtle features and weak distinctions required for beef freshness classification. Additionally, the limited capacity of shallow feature representations makes these models prone to insufficient extraction of critical information in the presence of complex background interference, resulting in a noticeable decline in overall classification performance. These quantitative results further demonstrate that DEVA-ConvNeXt surpasses existing models in Precision, Recall, and other key metrics for beef freshness grading.

**Figure 12:**
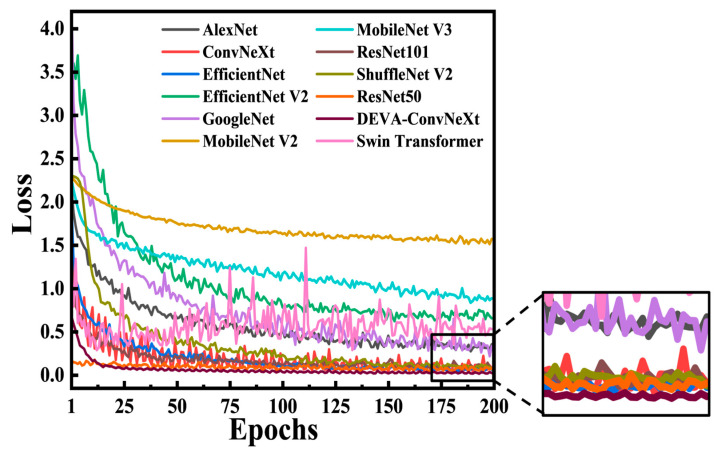
Comparison of validation loss between different models at different epochs.

To visually compare the validation loss across different models, Figure 12 presents a comparison of validation loss at different epochs for various models.

As shown in Figure 12, DEVA-ConvNeXt demonstrates superior loss performance on the validation set. The model rapidly decreases loss in the early stages and achieves quick convergence around the 25th epoch, maintaining a lower and more stable loss level. In contrast, traditional networks such as AlexNet and GoogLeNet exhibit higher and more fluctuating losses on the validation set, indicating limitations in their feature learning. The MobileNet series shows notably poorer loss performance. While ResNet and EfficientNet improve in convergence speed, their final stable loss values are still higher than those of DEVA-ConvNeXt, suggesting slower loss convergence compared to the proposed model.

To evaluate the performance of the improved model, three independent trials were conducted for both DEVA-ConvNeXt and ConvNeXt to ensure the reliability of the results. The findings show that DEVA-ConvNeXt significantly outperforms the baseline ConvNeXt across all key metrics, with stable results. For instance, DEVA-ConvNeXt achieved an accuracy of 94.8% ± 0.3%, which is about 6% higher than ConvNeXt’s 88.6% ± 0.4%, with a smaller standard deviation, ensuring result reliability. In terms of precision, recall, and F1 score, DEVA-ConvNeXt achieved 94.8% ± 0.2%, 94.6% ± 0.3%, and 94.7% ± 0.2%, respectively, all showing significant improvements over ConvNeXt’s 89.4% ± 0.2%, 88.7% ± 0.4%, and 88.7% ± 0.3%. The performance boost ranged from 5% to 6%. These results demonstrate that DEVA-ConvNeXt excels in both performance and stability, confirming the effectiveness of the improvements.

**Figure 13:**
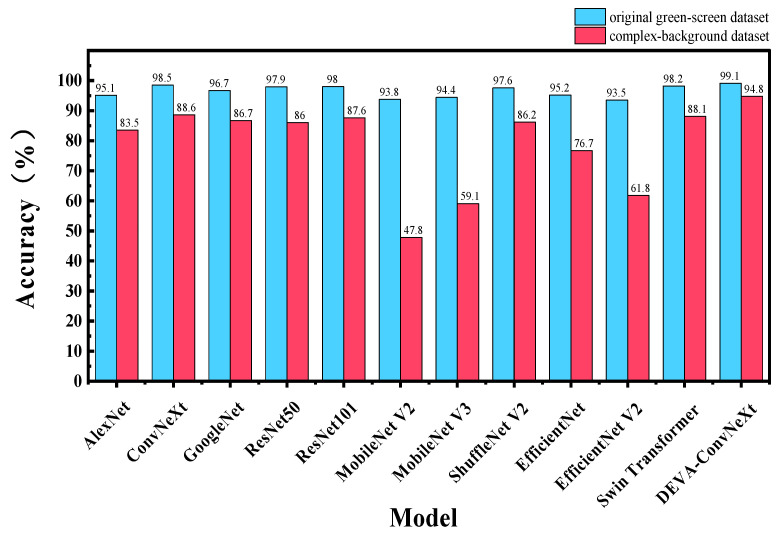
Accuracy performance of different models in beef freshness classification under single green screen and complex backgrounds.

To further validate the robustness of the DEVA-ConvNeXt model in complex environments, Figure 13 compares the performance of different models in beef freshness classification under green screen and complex backgrounds, emphasizing DEVA-ConvNeXt’s superiority in challenging settings.

As shown in Figure 13, DEVA-ConvNeXt’s accuracy dropped by 4.3% (from 99.1% to 94.8%) between the green screen and complex backgrounds, a much smaller decline compared to other models. For example, ConvNeXt’s accuracy dropped from 98.5% to 88.6%, a decrease of 9.9%, while MobileNet V2 and V3 experienced even larger accuracy drops in complex backgrounds. This comparison clearly shows that DEVA-ConvNeXt maintains high accuracy in complex settings, demonstrating its robustness in real-world beef freshness classification.

### 4.5. Visualization Performance Comparison of the DEVA-ConvNeXt Model

**Figure 14:**
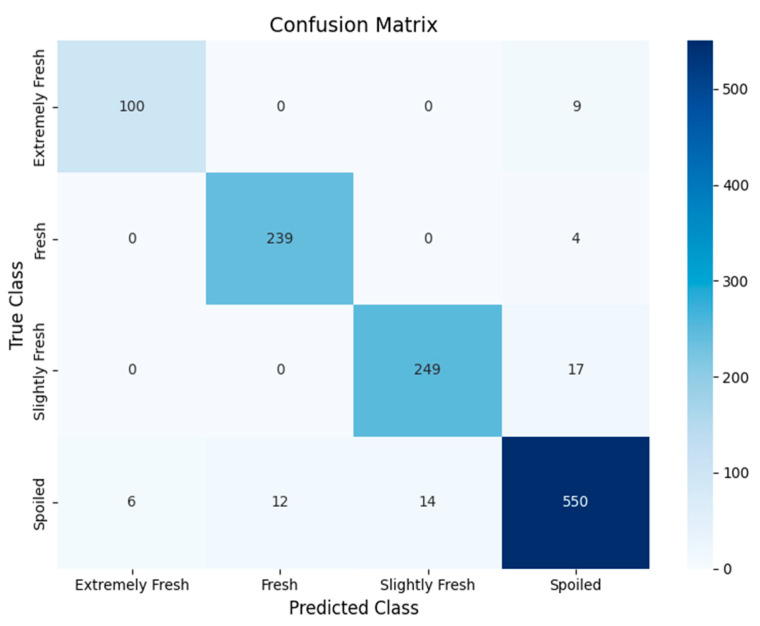
Confusion matrix of DEVA-ConvNeXt.

To visually demonstrate the performance of DEVA-ConvNeXt in classifying different freshness categories of beef, Figure 14 shows its confusion matrix.

As shown in Figure 14, the confusion matrix illustrates the relationship between the model’s predictions and the actual categories. The diagonal represents correctly classified samples, while the off-diagonal elements indicate misclassifications. The color depth reflects prediction accuracy. The model performs well in most categories. The “Spoiled” category has an accuracy of 94.83%, with 30 misclassified samples. The “Extremely Fresh” category has an accuracy of 98.75%, with 6 misclassified as “Spoiled.” The “Fresh” category has an accuracy of 98.67%, with 12 misclassified as “Spoiled.” The “Slightly Fresh” category has an accuracy of 97.42%, with 14 misclassified as “Spoiled.” Despite some misclassifications, the overall misclassification rate is low, indicating that the model effectively distinguishes between different freshness levels, especially between “Fresh” and “Extremely Fresh.” Overall, the model shows high accuracy, low misclassification, and stable, reliable performance.

**Figure 15:**
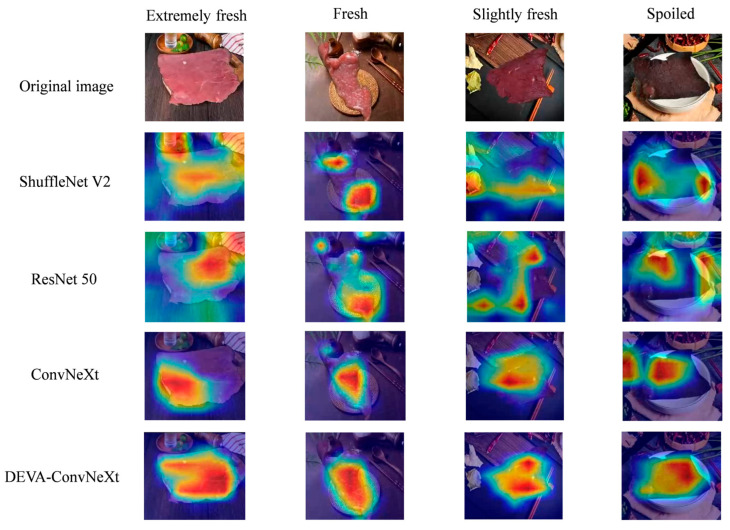
Heatmap results of DEVA-ConvNeXt and ConvNeXt.

To visually demonstrate the effectiveness of the information extracted by the DEVA-ConvNeXt model in the freshness grading task, this study applied the Grad-CAM visualization method. The results are shown in the figure. Grad-CAM uses different colors to indicate the sensitivity to image features, with red and yellow highlighting areas the model focuses on. The deeper the color, the more critical the features in that region. Figure 15 presents the heatmap visualization results of DEVA-ConvNeXt and ConvNeXt.

As shown in the heatmap results in Figure 15, it can be clearly observed that the attention regions of ShuffleNet V2 and ResNet-50 are relatively scattered, making them susceptible to interference from non-target areas in complex backgrounds and causing difficulty in consistently focusing on the meat portion. Compared with these two models, ConvNeXt performs better, as it is able to attend to the main meat region to some extent; however, it still struggles to suppress the influence of irrelevant factors. In contrast, the proposed DEVA-ConvNeXt model effectively suppresses background noise and directs its attention precisely and consistently toward the meat itself—the core subject of analysis—while minimizing the interference caused by the complex background According to the visualization in Figure 15, for extremely fresh, fresh, and slightly fresh samples, the proposed model primarily focuses on the surface of the entire piece of meat, indicating the model’s emphasis on evaluating global features such as its overall, uniform color and texture. For moderately spoiled and severely spoiled samples, the model’s attention is concentrated more on localized regions, accurately focusing on key points with darker color, decay, or abnormal texture. The heatmap visualization results further demonstrate the advantages of the proposed method in feature extraction and interference suppression.

### 4.6. Generalization Performance Evaluation

To validate the robustness and generalization of the proposed DEVA-ConvNeXt model across different datasets, we conducted a 5-fold cross-validation. The dataset was randomly divided into 10 parts: one for validation, one for testing, and the rest for training. A total of five experiments were performed to test beef freshness classification. The specific experimental groups are shown in Table 7. The average of these five experiments serves as both the model evaluation and an indicator of its error.

As shown in Table 7, the DEVA-ConvNeXt model achieves an average accuracy of 94.82%, precision of 95.02%, recall of 94.46%, and F1 score of 94.72% in the beef freshness classification task. The experimental data demonstrate that the model consistently performs with high accuracy across different validation sets, proving its generalizability.

**Figure 16:**
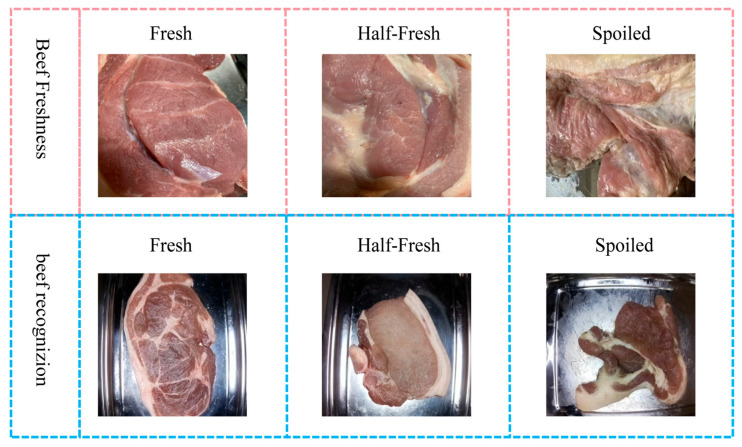
Sample images of beef freshness from different public datasets.

To assess the generalization capability of the DEVA-ConvNeXt model in beef freshness classification, cross-dataset tests were conducted on two public datasets from the Roboflow platform: Beef Freshness and beef recognizion. The Beef Freshness dataset contains 2266 images, categorized into Fresh, Half-Fresh, and Spoiled. The beef recognizion dataset consists of 3291 images, also classified into these three categories. Compared to the custom-built dataset, these public datasets show significant differences in data collection methods, camera angles, lighting variations, background complexity, and meat appearance, effectively testing the model’s generalization ability. Some sample data are shown below in Figure 16:

As shown in Table 8, the model generalization experiment results on different public datasets are provided in terms of Accuracy, Precision, Recall, and F1 Score.

As shown in Table 8, DEVA-ConvNeXt achieved 99.8% accuracy on the Beef Freshness dataset, with precision, recall, and F1 scores of 99.9%, 99.6%, and 99.7%, respectively. On the beef recognizion dataset, accuracy was 99.5%, with precision, recall, and F1 scores of 99.6%, 99.3%, and 99.4%. These results demonstrate that DEVA-ConvNeXt maintains high classification performance across publicly available datasets from different sources, confirming that its feature learning is not dependent on specific collection devices or conditions. The high performance in the generalization experiment further validates the model’s ability to generalize.

### 4.7. Performance Evaluation of the DEVA-ConvNeXt Model on Real Embedded Devices

**Figure 17:**
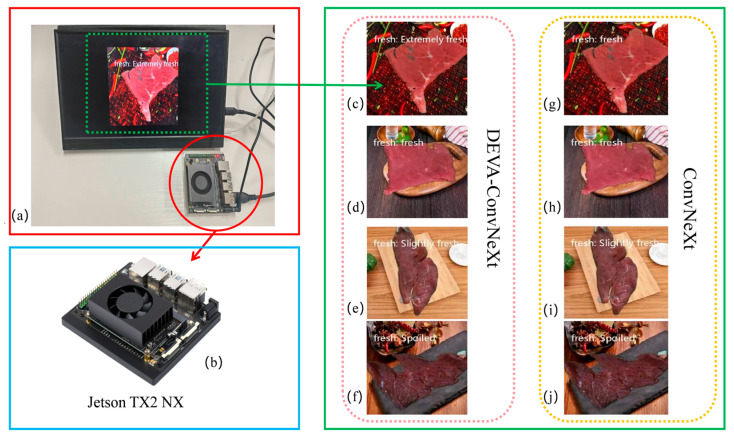
Visualization of testing results for beef freshness classification tasks on embedded platforms using different models. Panel (**a**) shows the actual embedded device and its display equipment; panel (**b**) zooms in on the embedded device highlighted by the red circle in panel (**a**). The green dashed line in panel (**a**) indicates the displayed content, while the green solid line box on the right presents the corresponding visual classification results. The classification results from the DEVA-ConvNeXt model are shown in the red dashed area within the green solid line box on the right (**c**–**f**), corresponding to the classification results for extremely fresh, fresh, slightly fresh, and spoiled samples, respectively. The classification results from the ConvNeXt model are displayed in the yellow dashed area within the green solid line box on the right (**g**–**j**), corresponding to the classification results for extremely fresh, fresh, slightly fresh, and spoiled samples, respectively.

To evaluate the real-time performance and feasibility of the DEVA-ConvNeXt model for beef freshness grading, the model was deployed on a Jetson TX2 NX device for testing. The Jetson TX2 features a dual-core NVIDIA Denver2 processor, a quad-core ARM Cortex-A57 CPU, and a 256-core NVIDIA Pascal GPU. The software environment includes Python 3.8 and PyTorch 1.8. The test set consists of 100 beef sample images with complex backgrounds, covering four different freshness levels. Figure 17 shows the visual test results of ConvNeXt and DEVA-ConvNeXt models in the beef freshness grading task on the embedded device.

As shown in (g) within the green solid box in Figure 17, ConvNeXt mistakenly classifies extremely fresh beef as fresh, mainly due to the similarity between the background and the beef’s color features. In contrast, DEVA-ConvNeXt performs better in both background interference and feature extraction, resulting in significantly better performance in freshness classification tasks. This is especially evident in tasks that require distinguishing between extremely fresh and fresh beef. In a deployment test with a set of 100 images, the baseline model ConvNeXt has a memory usage of 27.83 M, lower than DEVA-ConvNeXt’s 35.71 M. Additionally, its power consumption (21.658 W) on the Jetson TX2 NX is slightly lower than DEVA-ConvNeXt’s 23.927 W. As a result, DEVA-ConvNeXt has a slightly longer recognition time (0.02 s compared to ConvNeXt’s 0.015 s). In this independent deployment test, DEVA-ConvNeXt achieved an accuracy of 94%, higher than ConvNeXt’s 88%. To ensure reliability and statistical significance, we performed three independent repetitions of the test for both models, reporting the mean accuracy and standard deviation. The results show that DEVA-ConvNeXt achieved an accuracy of 94% ± 0.4%, while ConvNeXt reached 88% ± 0.3%, indicating that DEVA-ConvNeXt is more reliable in practical applications, particularly for accurate beef freshness classification, providing consistent and precise results.

From a practical application perspective, DEVA-ConvNeXt shows great potential in the development and use of beef freshness grading equipment. Its strong feature extraction and anti-interference capabilities enable accurate freshness judgment even in complex backgrounds. In food quality detection, DEVA-ConvNeXt offers significant advantages, especially in the automated systems for detection of beef freshness, effectively improving detection precision, reducing manual intervention, and enhancing overall efficiency.

## 5. Conclusions

This study addresses the challenges of background interference and feature extraction difficulties in beef freshness grading tasks under complex backgrounds. We propose the DEVA-ConvNeXt model, which introduces a dynamic non-local coordinate attention mechanism (DNLC) and an enhanced deep convolution module (EDW). These innovations significantly improve the ConvNeXt network’s ability to extract deep features and perform multi-scale fusion, effectively mitigating the impact of complex backgrounds. Additionally, we use Varifocal Loss as an optimization strategy to enhance the model’s ability to learn subtle feature differences and speed up training convergence. Combined with the TVB-N metric, this enables precise freshness grading of the dataset. To address low data collection efficiency and improve robustness in complex environments, we propose the novel Alpha-Background Generation Shift (ABG-Shift) background replacement technique, creating a beef freshness dataset under complex backgrounds and enhancing the model’s generalization ability. Extensive experiments show that DEVA-ConvNeXt achieves Accuracy, Precision, Recall, and F1 scores of 94.8%, 94.8%, 94.6%, and 94.7%, respectively, outperforming ConvNeXt by 6.2%, 5.4%, 5.9%, and 6.0%. It also surpasses classic models like ResNet101 and ShuffleNet V2 in all metrics. Testing on embedded devices demonstrates the model’s capability to accurately assess beef freshness by analyzing key features like texture and color. This verifies the practical feasibility of the proposed solution for beef freshness grading devices. The current study provides a foundation for intelligent food quality detection, but it has limitations in real-time performance and computational efficiency, particularly on resource-constrained embedded devices. Addressing these limitations is crucial for improving grading accuracy and reliability. Future research will prioritize optimizing performance on embedded systems to ensure scalability. We also aim to broaden the scope by developing methods to grade the freshness of various meat types, offering a holistic and non-destructive food quality detection solution.

## Figures and Tables

**Table 1:** TVB-N content-based grading of beef freshness.

Meat Freshness Grading	Volatile Basic Nitrogen Content (mg/100 g)
Grade 1 Freshness (Fresh)	≤15
Grade 2 Freshness (Slightly Fresh)	15–25
Spoiled	≥25

**Table 2:** Statistical distribution of the beef freshness sample image dataset.

Freshness Grade	Days	Quantity
Extremely fresh	0	109
Fresh	1	123
	2	120
Slightly fresh	3	137
	4	129
Spoiled	5	118
	6	125
	7	130
	8	110
	9	99

**Table 3:** Experimental parameters configuration.

Parameter	Value
Epochs	200
Batch size	8
Image size	224 × 224
Optimizer algorithm	Adam
Learning rate	0.0006
Weight decay	0.01

**Table 4:** Performance comparison of different attention mechanisms in beef freshness grading under complex backgrounds.

Model	Accuracy (%)	Precision (%)	Recall (%)	F1 Score (%)
SE	89.6	90.7	89.2	89.9
SimAM	89.3	90.5	89.0	89.7
CBAM	89.9	91.0	89.4	90.1
CA	90.7	91.5	90.5	90.9
DNLC	92.8	92.8	92.8	92.8

**Table 5:** Ablation study results for evaluating model performance improvements.

ConvNeXt	DNLC	EDW	VF Loss	Accuracy (%)	Precision (%)	Recall (%)	F1 Score (%)
√				88.6	89.6	88.9	88.9
√	√			92.8	92.8	92.8	92.8
√		√		90.2	90.6	90.3	90.6
√			√	88.4	89.3	88.8	89.0
√	√	√		94.7	94.8	94.5	94.6
√	√	√	√	94.8	94.8	94.6	94.7

**Table 6:** Performance comparison of different models.

Model	Accuracy (%)	Precision (%)	Recall (%)	F1 Score (%)
AlexNet	83.5	82.8	83.2	83.5
ConvNeXt	88.6	89.4	88.7	88.7
GoogleNet	86.7	87.4	86.4	86.7
ResNet50	86.0	86.4	86.0	86.1
ResNet101	87.6	88.1	87.4	87.7
MobileNet V2	47.8	48.5	47.3	47.8
MobileNet V3	59.1	59.5	58.8	59.1
ShuffleNet V2	86.2	86.6	85.9	86.2
EfficientNet	76.7	77.1	76.7	76.8
EfficientNet V2	61.8	62.3	61.8	62.0
Swin Transformer	88.1	88.4	88.1	88.2
DEVA-ConvNeXt	94.8	94.8	94.6	94.7

**Table 7:** Results of the 5-fold cross-validation experiment for the DEVA-ConvNeXt model.

Group	Train Set	Val Set	Test Set	Accuracy (%)	Precision (%)	Recall (%)	F1 Score (%)
1	1, 2, 3, 4, 5, 6, 7, 8	10	9	95.2	95.5	95.0	95.2
2	2, 3, 4, 5, 6, 7, 8, 10	1	9	94.4	94.1	94.6	94.3
3	1, 3, 4, 5, 6, 7, 8, 10	2	9	94.9	95.3	94.1	94.7
4	1, 2, 4, 5, 6, 7, 8, 10	3	9	95.1	95.4	94.6	95.0
5	1, 2, 3, 5, 6, 7, 8, 10	4	9	94.5	94.8	94.0	94.4

**Table 8:** Model generalization experiment results on different public datasets.

Model	Dataset	Accuracy (%)	Precision (%)	Recall (%)	F1 Score (%)
DEVA-ConvNeXt	Beef Freshness	99.8	99.9	99.6	99.7
	beef recognizion	99.5	99.6	99.3	99.4

## Data Availability

The original contributions presented in the research are included in the article; further inquiries can be directed to the corresponding author.

## References

[1] Khalid W., Maggiolino A., Kour J., Arshad M.S., Aslam N., Afzal M.F., Meghwar P., Zafar K.-u.-W., De Palo P., Korma S.A. (2023). Dynamic alterations in protein, sensory, chemical, and oxidative properties occurring in meat during thermal and non-thermal processing techniques: A comprehensive review. Front. Nutr..

[2] Bekhit A.E.-D.A., Holman B.W., Giteru S.G., Hopkins D.L. (2021). Total volatile basic nitrogen (TVB-N) and its role in meat spoilage: A review. Trends Food Sci. Technol..

[3] Wang B., Yang H., Yang C., Lu F., Wang X., Liu D. (2022). Prediction of total volatile basic nitrogen (TVB-N) and 2-thiobarbituric acid (TBA) of smoked chicken thighs using computer vision during storage at 4 °C. Comput. Electron. Agric..

[4] Zuo J., Peng Y., Li Y., Yin T., Chao K. (2024). Nondestructive intelligent detection of total viable count in pork based on miniaturized spectral sensor. Food Res. Int..

[5] Shtepliuk I., Domènech-Gil G., Almqvist V., Kautto A.H., Vågsholm I., Boqvist S., Eriksson J., Puglisi D. (2025). Electronic nose and machine learning for modern meat inspection. J. Big Data.

[6] Jia W., Lv H., Liu Y., Zhou W., Qin Y., Ma J. (2024). Automated detection of stale beef from electronic nose data. Food Sci. Nutr..

[7] Shin S., Lee Y., Kim S., Choi S., Kim J.G., Lee K. (2021). Rapid and non-destructive spectroscopic method for classifying beef freshness using a deep spectral network fused with myoglobin information. Food Chem..

[8] Arias E., Sierra V., Prado N., González P., Fiorentini G., Díaz J., Oliván M. (2022). Development of a portable near-infrared spectroscopy tool for detecting freshness of commercial packaged pork. Foods.

[9] Elangovan P., Dhurairajan V., Nath M.K., Yogarajah P., Condell J. (2024). A novel approach for meat quality assessment using an ensemble of compact convolutional neural networks. Appl. Sci..

[10] Huang H., Zhan W., Du Z., Hong S., Dong T., She J., Min C. (2022). Pork primal cuts recognition method via computer vision. Meat Sci..

[11] Song S., Guo Q., Duan X., Shi X., Liu Z. (2024). Research on Pork Cut and Freshness Determination Method Based on Computer Vision. Foods.

[12] Liu C., Zhang J., Chen K., Huang J. (2025). BBSNet: An Intelligent Grading Method for Pork Freshness Based on Few-Shot Learning. Foods.

[13] Syaukani I., Muji S.Z.B.M., Kan C.U.E. (2025). Classification of Beef and Pork Using a Hybrid Model of ResNet-50 and Support Vector Machine (SVM). J. Sci. Technol. Innov..

[14] Xi R., Lyu X., Yang J., Lu P., Duan X., Hopkins D.L., Zhang Y. (2025). Non-Destructive and Real-Time Discrimination of Normal and Frozen-Thawed Beef Based on a Novel Deep Learning Model. Foods.

[15] Singh R., Nickhil C., Nisha R., Upendar K., Jithender B., Deka S.C. (2025). A comprehensive review of advanced deep learning approaches for food freshness detection. Food Eng. Rev..

[16] Niu L., Cong W., Liu L., Hong Y., Zhang B., Liang J., Zhang L. (2021). Making images real again: A comprehensive survey on deep image composition. arXiv.

[17] Gat N., Subramanian S., Barhen J., Toomarian N. Spectral imaging applications: Remote sensing, environmental monitoring, medicine, military operations, factory automation, and manufacturing. Proceedings of the 25th AIPR Workshop: Emerging Applications of Computer Vision.

[18] Holman B.W., Bekhit A.E.-D.A., Waller M., Bailes K.L., Kerr M.J., Hopkins D.L. (2021). The association between total volatile basic nitrogen (TVB-N) concentration and other biomarkers of quality and spoilage for vacuum packaged beef. Meat Sci..

[19] (2016). National Food Safety Standard for Fresh (Frozen) Meat and Poultry Products.

[20] (2016). Determination of Total Volatile Basic Nitrogen in Foods.

[21] Tew Y., Lee W.Y., Tam G.M. Parallel Computing Using CUDA and MultiThreading in Background Removal Process. Proceedings of the 2024 3rd International Conference on Digital Transformation and Applications (ICDXA).

[22] Liu Z., Mao H., Wu C.-Y., Feichtenhofer C., Darrell T., Xie S. A convnet for the 2020s. Proceedings of the 2022 IEEE/CVF Conference on Computer Vision and Pattern Recognition.

[23] Zhang H., Wang Y., Dayoub F., Sunderhauf N. Varifocalnet: An IoU-aware dense object detector. Proceedings of the 2021 IEEE/CVF Conference on Computer Vision and Pattern Recognition.

[24] Mao A., Mohri M., Zhong Y. Cross-entropy loss functions: Theoretical analysis and applications. Proceedings of the 40th International Conference on Machine Learning.

[25] Liu Z., Lin Y., Cao Y., Hu H., Wei Y., Zhang Z., Lin S., Guo B. Swin transformer: Hierarchical vision transformer using shifted windows. Proceedings of the 2021 IEEE/CVF International Conference on Computer Vision.

[26] Chollet F. Xception: Deep learning with depthwise separable convolutions. Proceedings of the 2017 IEEE Conference on Computer Vision and Pattern Recognition.

[27] (2022). Grading of Livestock and Poultry Meat Quality.

[28] Hua B.-S., Tran M.-K., Yeung S.-K. Pointwise convolutional neural networks. Proceedings of the IEEE Conference on Computer Vision and Pattern Recognition.

[29] Hu J., Shen L., Sun G. Squeeze-and-excitation networks. Proceedings of the IEEE Conference on Computer Vision and Pattern Recognition.

[30] Yang L., Zhang R.-Y., Li L., Xie X. SimAM: A simple, parameter-free attention module for convolutional neural networks. Proceedings of the International Conference on Machine Learning.

[31] Woo S., Park J., Lee J.-Y., Kweon I.S. CBAM: Convolutional block attention module. Proceedings of the European Conference on Computer Vision (ECCV).

[32] Hou Q., Zhou D., Feng J. Coordinate attention for efficient mobile network design. Proceedings of the 2021 IEEE/CVF Conference on Computer Vision and Pattern Recognition.

[33] Krizhevsky A., Sutskever I., Hinton G.E. (2012). Imagenet classification with deep convolutional neural networks. Neural Inf. Process. Syst..

[34] Koonce B. (2021). ResNet 50. Convolutional Neural Networks with Swift for Tensorflow: Image Recognition and Dataset Categorization.

[35] Szegedy C., Liu W., Jia Y., Sermanet P., Reed S., Anguelov D., Erhan D., Vanhoucke V., Rabinovich A. Going deeper with convolutions. Proceedings of the 2015 IEEE Conference on Computer Vision and Pattern Recognition.

[36] Sandler M., Howard A., Zhu M., Zhmoginov A., Chen L.-C. MobileNetV2: Inverted residuals and linear bottlenecks. Proceedings of the 2018 IEEE Conference on Computer Vision and Pattern Recognition.

[37] Howard A., Sandler M., Chu G., Chen L.-C., Chen B., Tan M., Wang W., Zhu Y., Pang R., Vasudevan V. Searching for MobileNetV3. Proceedings of the 2019 IEEE/CVF International Conference on Computer Vision.

[38] Tan M., Le Q. EfficientNet: Rethinking model scaling for convolutional neural networks. Proceedings of the International Conference on Machine Learning.

[39] Tan M., Le Q. EfficientNetV2: Smaller models and faster training. Proceedings of the 38th International Conference on Machine Learning.

[40] Ma N., Zhang X., Zheng H.-T., Sun J. ShuffleNet V2: Practical guidelines for efficient CNN architecture design. Proceedings of the European Conference on Computer Vision (ECCV).

